# Data characterizing the chloroplast genomes of extinct and endangered Hawaiian endemic mints (Lamiaceae) and their close relatives

**DOI:** 10.1016/j.dib.2016.03.037

**Published:** 2016-03-14

**Authors:** Andreanna J. Welch, Katherine Collins, Aakrosh Ratan, Daniela I. Drautz-Moses, Stephan C. Schuster, Charlotte Lindqvist

**Affiliations:** a Department of Biological Sciences, University at Buffalo (SUNY), Buffalo, NY 14260, USA; b Department of Public Health Sciences and Center for Public Health Genomics, University of Virginia, Charlottesville 22908, VA, USA; c Singapore Centre on Environmental Life Sciences Engineering, Nanyang Technological University, Singapore 637551, Singapore

**Keywords:** Hawaii, Lamiaceae, Plastid genomes, Genome structure

## Abstract

These data are presented in support of a plastid phylogenomic analysis of the recent radiation of the Hawaiian endemic mints (Lamiaceae), and their close relatives in the genus *Stachys*, “The quest to resolve recent radiations: Plastid phylogenomics of extinct and endangered Hawaiian endemic mints (Lamiaceae)” [1]. Here we describe the chloroplast genome sequences for 12 mint taxa. Data presented include summaries of gene content and length for these taxa, structural comparison of the mint chloroplast genomes with published sequences from other species in the order Lamiales, and comparisons of variability among three Hawaiian taxa vs. three outgroup taxa. Finally, we provide a list of 108 primer pairs targeting the most variable regions within this group and designed specifically for amplification of DNA extracted from degraded herbarium material.

**Specifications Table**

**Table:** 

Subject area	*Biology, genetics, genomics*
More specific subject area	*Molecular phylogenetics and evolution*
Type of data	*Tables and figures*
How data was acquired	*High-throughput sequencing of contemporary and herbarium samples was conducted on the Illumina HiSeq 2500 and MiSeq platforms, followed by both de novo and reference-guided assemblies, mapping, and functional annotation*
Data format	*Raw, and analyzed*
Experimental factors	*De novo assemblies were created using SOAPdenovo and reference-guided assemblies were created using YASRA. Sequences were functionally annotated using DOGMA. SNPs were called and filtered using SAMtools and BCFtools*
Experimental features	*Data include chloroplast genome gene content, structure, and comparisons of variable loci in a suite of recently diverged species and outgroups*
Data source location	*Hawaii, North America, South America, Europe, Africa, and Asia*
Data accessibility	*Data are published with this article*

**Value of the data**

• These data provide a summary of the characteristics and structure of the chloroplast genomes of several taxa within Lamiaceae, which can be used to increase our understanding of molecular evolution of the chloroplast genome, as well as the evolution of its structure and function.
• A comparison of variable regions in mints can be used to identify rapidly evolving regions in other taxa.
• Primer sequences described here can be used to target highly variable regions in closely related taxa.

## 1 Data

Raw, demultiplexed sequence reads have been deposited in the NCBI sequence read archive (SRP070171) and full chloroplast genomes for 12 mint taxa have been deposited in GenBank (KU724130-KU724141). Data presented in the text include tables and figures giving information on gene content and variability in these 12 species, as well as comparison of genome structure with other members of the order Lamiales.

## 2 Experimental design, materials and methods

### 2.1 Samples, library construction, and shotgun sequencing

We selected 12 Hawaiian mint taxa for shotgun sequencing (five contemporary and seven from herbarium collections ranging up to ~100 years old), of which two extinct species were represented by two accessions each (see Tables 1 and 2 in [1]). We also sequenced four *Stachys* species, representing both close and more distantly related relatives.

DNA extraction, library construction and shotgun sequencing followed the methods described in [1]. Briefly, approximately 100 mg dried leaf tissue was homogenized using the TissueLyser system (Qiagen), and DNA was extracted using the DNeasy plant mini kit (Qiagen). DNA isolated from herbarium samples was processed separately from contemporary samples using stringent protocols and controls to prevent and detect any potential contamination. For contemporary samples, DNA extracts were sheared to 200–600 bp via sonication in a Covaris S220; DNA from herbarium samples is naturally degraded and therefore was not sheared further. Genomic shotgun sequencing libraries were constructed following the standard Illumina Tru-seq protocol for contemporary samples, or the NEBNext Library Prep Mastermix kit (New England Biolabs) for herbarium samples. Libraries were quantified using the PicoGreen High Sensitivity assay and then pooled and sequenced on the Illumina HiSeq and MiSeq platforms. Adapter sequences were trimmed from the reads using the AdapterRemoval software [2]. Assessment of DNA damage in old herbarium specimens was conducted using mapDamage 2.0 [3]. The presence of misincorporations characteristic of damaged DNA molecules typically found in old and degraded samples suggests that the data from herbarium samples are authentic, however, the overall levels of damage were low and within the range expected based on the age of the specimens (see Supplementary Figs. 2 and 3 in [1]).

### 2.2 Assembly of the Hawaiian mint reference chloroplast genome

Because no chloroplast genome sequence from a closely related taxon was available at the time this study was conducted, we implemented a combined reference-guided and *de novo* assembly approach [4] to determine the first complete chloroplast genome sequence for a Hawaiian mint. We assembled the sequence for *Stenogyne haliakalae*, an extinct species, as it had the largest number of reads. Briefly, the approach involved conducting both reference-guided assembly in YASRA 2.32 [5] with olive (*Olea europaea*, NC_013707; [6]) as the reference, as well as *de novo* assembly in SOAPdenovo v1.05 [7]. Assembly methods are described in more detail in [1]. The resulting contigs from both approaches were split into overlapping sequences, and then used as input for a further reference guided-assembly step in YASRA. Gaps between the final contigs were closed using PCR (see [1] for PCR reaction conditions and Supplementary Table 1 of this paper for primer information) and Sanger sequencing in both directions from high-quality DNA extracted from a contemporary sample of *Stengyne bifida*. This ensured that amplification could be carried out over potentially large gaps, which would not be possible with degraded DNA from the extinct *Stenogyne haliakalae*. Contigs and Sanger sequences were aligned in Sequencher 4.7 (Gene Codes) to create a pseudo-reference sequence [4]. Reads from *Stenogyne haliakalae* were then mapped to the pseudo-reference using BWA v. 0.6.2 [8]. The reference sequence was further refined through Sanger sequencing of areas with low coverage or poor mapping quality (e.g., the border between the inverted repeat and single copy region). Reads were mapped to the final sequence, PCR duplicates were flagged and removed with the MarkDuplicates tool of the Picard command line toolset (http://picard.sourceforge.net/index.shtml), and a consensus sequence was called using SAMtools [9].

### 2.3 Assembly of additional mint chloroplast genomes

Complete or nearly complete chloroplast genomes were assembled using similar methods for 11 additional taxa: seven from the endemic Hawaiian mints (two of which were from herbarium samples) and four *Stachys* outgroups (see Tables 1 and 2 in [1]). Since the Hawaiian mints have diverged recently, we used the new chloroplast genome sequence from *Stenogyne haliakalae* as the reference during reference-guided assembly for the remaining Hawaiian taxa. The resulting contigs were aligned to create an interim sequence, and then the reads were mapped to this using BWA and a final consensus sequence called using SAMtools.

Chloroplast genome sequences for the *Stachys* outgroup taxa were assembled in a similar manner. We first assembled the chloroplast genome sequence for *Stachys chamissonis*, as this species is most closely related to the Hawaiian lineage. We conducted independent YASRA runs using *Olea europaea* as the reference, in addition to newly available sequences from *Stenogyne haliakalae*, *Sesamum indicum* (NC_016433) [10], *Origanum vulgare* (JX880022) [11], and *Salvia miltiorrhiza* (NC_020431) [12]. The contigs from all five runs were aligned to create an interim sequence. The reads were then mapped to the interim sequence using BWA and a consensus called using SAMtools. Once the *Stachys chamissonis* sequence was assembled we used this as the reference in YASRA for reference guided assembly of both *Stachys coccinea* and *Stachys sylvatica*. For *Stachys byzantina*, the most distantly related outgroup, we performed the initial reference-guided assembly using the sequence from *Stachys chamissonis*, as well as *Olea europaea* and *Sesamum indicum*. The rest of the assembly proceeded as described for the other *Stachys* species.

### 2.4 Gene content and structure of mint chloroplast genomes

The *Stenogyne haliakalae* reference sequence and sequences from additional species were annotated using a combination of DOGMA [13], tRNAscan-SE [14], and additional manual BLAST searches. The borders of the inverted repeats were identified with the program Inverted Repeats Finder [15].

Overall the chloroplast genome sequences assembled here are very similar to other Lamiales. Table 1 shows the gene content of the *Stenogyne haliakalae* chloroplast genome, which mirrors the gene content of the chloroplast genomes for the 11 additional mint taxa, including the *Stachys* outgroups. Table 2 lists the genes that contain introns (and the number of introns present) in the mint taxa investigated here. Table 3 gives the lengths of the full chloroplast genome for each sequence assembled, as well as the lengths of the inverted repeats and single copy regions.

**Fig. 1:**
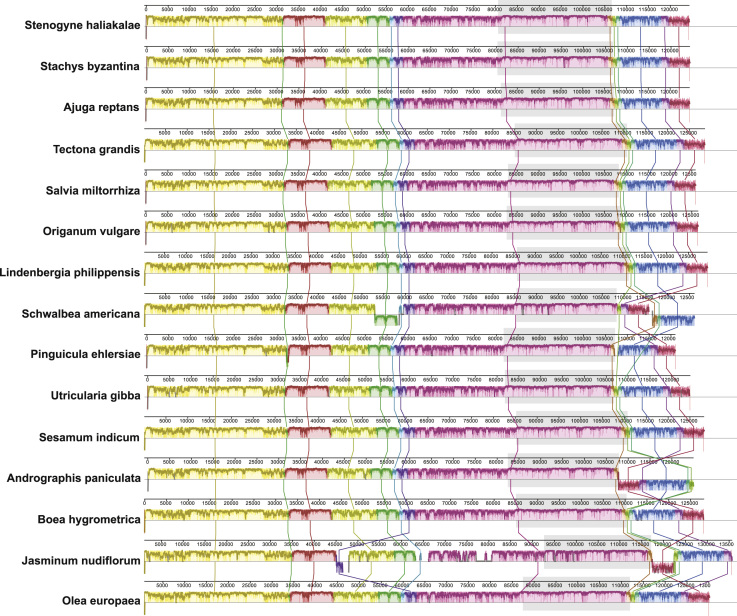
Comparison of structure and similarity among 15 complete chloroplast genomes from the order Lamiales**.***Cistanche deserticola* (102,657 bp) and *Epifagus virginiana* (70,028 bp), both members of the Orobanchaceae, are not considered here because they are parasitic and lack chlorophyll, thus demonstrating largely reduced chloroplast genomes. Blocks with the same color represent homologous regions free of internal structural changes for that subset of taxa, and those above the centerline for each taxon are in the same orientation as in *Stenogyne haliakalae*, whereas those below the line are in the reverse direction. Within each block a similarity profile for the region is plotted. Areas outside of blocks are presumed to represent lineage-specific regions of the chloroplast genome. One copy of the inverted repeat has been trimmed so that homology of the remaining repeat (area shaded in light gray) can be shown.

To compare the genome structure of the Hawaiian and *Stachys* taxa to other taxa in the order Lamiales, we conducted analyses in Mauve 2.3.1 [16] (Fig. 1). In the analysis we included *Stenogyne haliakalae, Stachys byzantina, Ajuga reptans* (NC_023102), *Andrographis paniculata* (NC_022451), *Boea hygrometrica* (NC_016468), *Jasminum nudiflorum* (NC_008407), *Lindenbergia philippensis* (NC_022859), *Olea europaea* (NC_013707), *Origanum vulgare* (JX880022), *Pinguicula ehlersiae* (NC_023463), *Salvia miltiorrhiza* (NC_020431), *Schwalbea americana* (NC_023115), *Sesamum indicum* (NC_016433), *Tectona grandis* (NC_020098), and *Utricularia gibba* (NC_021449). The chloroplast genome sequences of some taxa in this analysis demonstrated rearrangements and inversions. Therefore, one of the inverted repeats was trimmed off at the coordinates suggested by Inverted Repeats Finder so that homology could be determined with the remaining region. Seed weight was set to 19, the gap opening penalty was set to −200, and the gap extension penalty to −30.

**Fig. 2:**
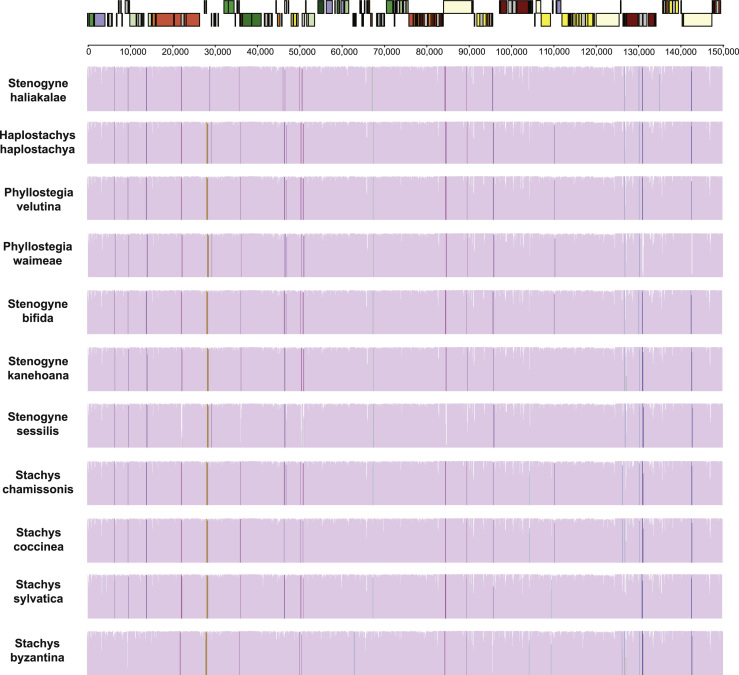
Conservation among 11 complete mint chloroplast genomes**.** The sequence for *Haplostachys linearifolia* was excluded due to missing data. A physical map is given at the top to show gene content and organization (see Fig. 2 in [1] for gene names and products). In the lower panels, regions of the genome are represented by bars, and those that are conserved among all 11 species are colored mauve, whereas those that are conserved among subsets of the taxa have different colors. The height of the bar shows the degree of similarity.

To compare the genome structure of all of the Hawaiian and *Stachys* mints to each other, we conducted a separate analysis in Mauve (Fig. 2). The sequences were assumed to be collinear. The seed weight was set to 7, the gap opening penalty was set to −200, and the gap extension penalty to −30.

### 2.5 Variability in the chloroplast genome sequences of Hawaiian mints and *Stachys* outgroups

We investigated variability among the three highest quality chloroplast genome sequences of the Hawaiian mints (*Stenogyne haliakalae, Stenogyne bifida, Haplostachys haplostachya*), and the three highest quality sequences from the *Stachys* outgroups (*Stachys chamissonis, Stachys coccinea,* and *Stachys sylvatica*). These species represent all of the main lineages within our samples, and were sequenced at >10× depth. We used BWA to map the reads for each of these species onto the *Stenogyne haliakalae* reference genome and used SAMtools and BCFtools to call SNPs with a SNP quality score >30. Annotations of the *S. haliakalae* reference genome were transferred to the locations of the SNPs. We compared the levels of chloroplast genome diversity among the six genomes by identifying unique, variable positions, which we refer to as potentially informative characters (PICs) [17], [18]. We did not include indels and inversions in this definition, which have been included in other analyses of chloroplast genome variability.

**Fig. 3:**
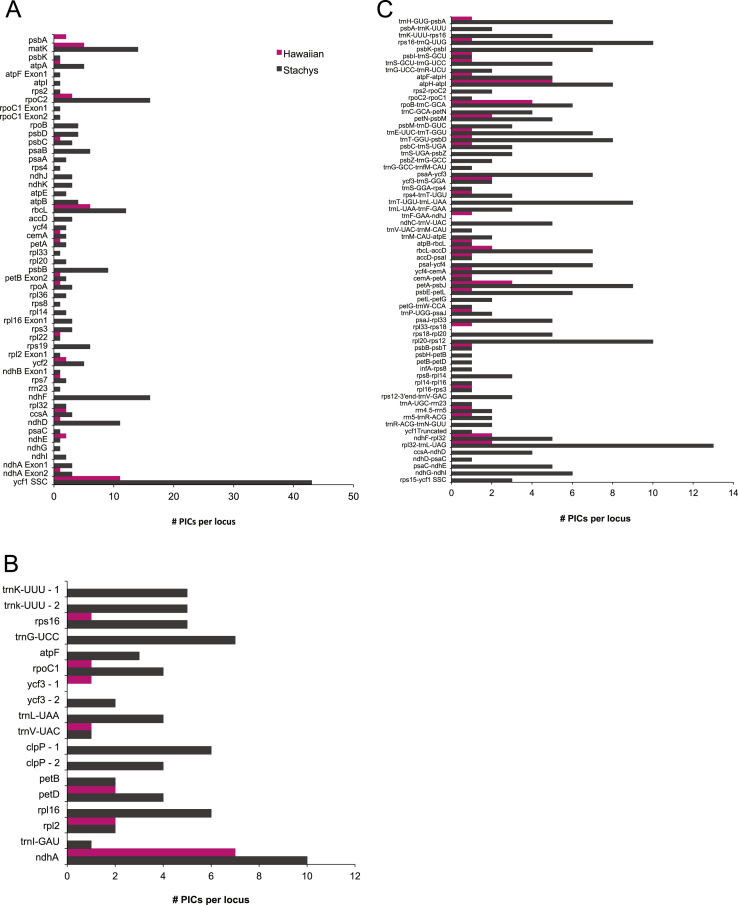
Comparison of the number of potentially informative characters (PICs) per locus in (a) protein coding regions, (b) introns, and (c) intergenic spacers and pseudogenes for chloroplast genomes of three Hawaiian and three *Stachys* taxa: *Stenogyne haliakalae, Stenogyne bifida, Haplostachys Haplostachya, Stachys chamissonis, Stachys coccinea,* and *Stachys sylvatica*. Regions that were completely conserved among these species and/or that are shorter than 100 bp are not shown. Pink=three Hawaiian taxa; Black=three *Stachys* taxa. (For interpretation of the references to color in this figure legend, the reader is referred to the web version of this article.)

**Fig. 4:**
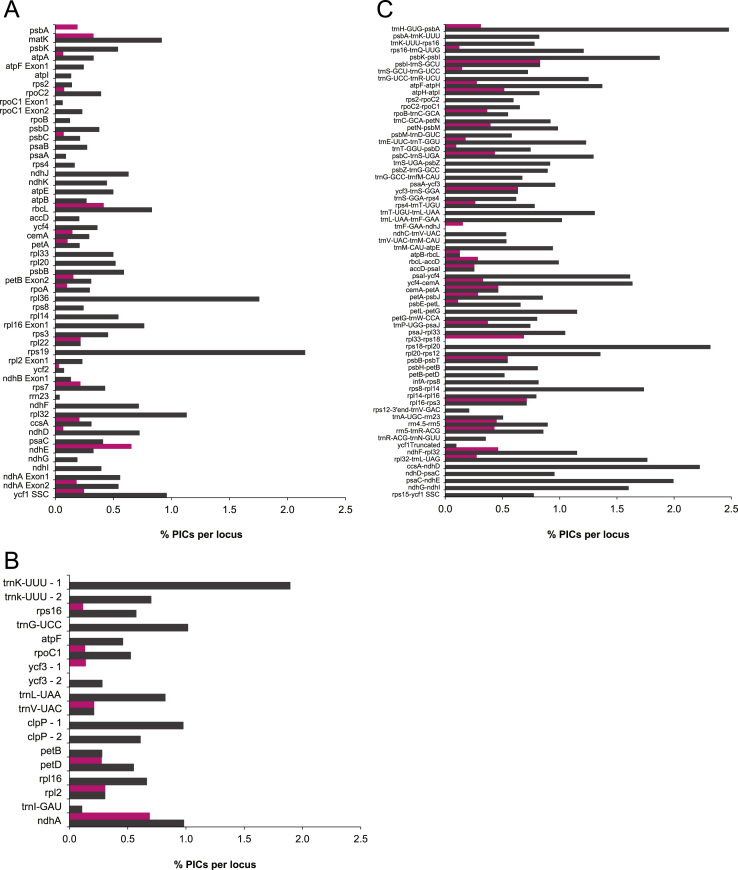
Comparison of the % PICs (potentially informative characters) per locus in (a) protein coding regions, (b) introns, and (c) intergenic spacers and pseudogenes for chloroplast genomes of three Hawaiian and three *Stachys* taxa: *Stenogyne haliakalae, Stenogyne bifida, Haplostachys haplostachya, Stachys chamissonis, Stachys coccinea,* and *Stachys sylvatica*. Regions that were completely conserved among these species or that are shorter than 100 bp are not shown. Pink=three Hawaiian taxa; Black=three *Stachys* taxa. (For interpretation of the references to color in this figure legend, the reader is referred to the web version of this article.)

To analyze diversity among the chloroplast genomes, we compared the number of PICs present in 1000 bp non-overlapping sliding windows across the entire chloroplast genome sequences (Table 4, see also Fig. 5 in [1]). We also compared the number of PICs per locus for coding (Table 5, Fig. 3a), intron (Table 6, Fig. 3b), and intergenic spacer and pseudogene regions (Table 7, Fig. 3c). Because this approach does not take into account the length of the locus, very long loci appear to have more PICs than shorter loci. Therefore, we also divided the number of PICs by the total length of the region to give the percent PICs per locus (Table 5, Table 6, Table 7, Fig. 4). However, very short regions may still appear to have a high percentage of variable sites, when in fact only a small number of the sites were variable. To minimize this, we have excluded regions less than 100 bp in length, and for clarity we have also excluded regions that were conserved among all six taxa.

To identify the most variable regions of the mint chloroplast genome for targeted re-sequencing and high resolution phylogenetic analyzes, reads from all 15 taxa subjected to shotgun sequencing (including the partial genomes from all of the historical samples, except *Phyllostegia variabilis*) were mapped to the *Stenogyne haliakalae* reference sequence using BWA. SNPs were called with SAMtools and BCFtools, filtering out those with SNP quality <30. We selected a total of 108 variable loci (see Fig. 2 in [1] for a diagram of locations) identified from single copy regions, including (1) all the regions that had a variant position among the Hawaiian mints (except where every individual had an alternative allele as compared to the reference sequence) and (2) additional regions that had variant positions among at least two of the *Stachys* species. 100 bp of flanking sequence on either side of the SNPs was retrieved from the reference genome, and PCR primers were designed using BatchPrimer3 [19], with further manual examination for quality control (e.g. to ensure that primer sequences did not fall into a gap for one of the other taxa). Sequences complementary to the Illumina sequencing adapters were appended to the end of each primer (Table 8) so that sequencing libraries could be prepared directly from the cleaned multiplex PCR products. Overall, these regions represent roughly 20,000 bp of sequence from the chloroplast genome and contain additional variable sites beyond the initial targeted SNP.

## Figures and Tables

**Table 1:** Gene content of the chloroplast genome of *Stenogyne haliakalae* and 11 additional mint species.

**Gene Products**	**Genes**
Photosystem I	psaA, psaB, psaC, psaI, psaJ, ycf3 [20], ycf4 [20]
Photosystem II	psbA, psbB, psbC, psbD, psbE, psbF, psbH, psbI, psbJ, psbK, psbL, psbM, psbN, psbT, psbZ/lhbA
Cytochrome b6/f	petA, petB, petD, petG, petL, petN
ATP synthase	atpA, atpB, atpE, atpF, atpH, atpI
Rubisco	rbcL
NADH oxidoreductase	ndhA, ndhB^a^, ndhC, ndhD, ndhE, ndhF, ndhG, ndhH, ndhI, ndhJ, ndhK
RNA polymerase	rpoA, rpoB, rpoC1, rpoC2
Large subunit ribosomal proteins	rpl2^a^, rpl14, rpl16, rpl20, rpl22, rpl23^a^, rpl32, rpl33, rpl36
Small subunit ribosomal proteins	rps2, rps3, rps4, rps7^a^, rps8, rps11, rps12^b^, rps14, rps15, rps16, rps18, rps19
Other functions	accD, ccsA, cemA, clpP, matK, infA
Unknown functions	ycf1^b^, ycf2^a^, ycf15^a^
Ribosomal RNAs	rrn23^a^, rrn16^a^, rrn5^a^, rrn4.5^a^
Transfer RNAs	trnA(UGC)^a^, trnC(GCA), trnD(GUC), trnE(UUC), trnF(GAA), trnG(GCC), trnG(UCC), trnH(GUG), trnI(CAU)^a^, trnI(GAU)*, trnK(UUU), trnL(UAA), trnL(UAG), trnL(CAA)^a^, trnfM(CAU), trnM(CAU), trnN(GUU)^a^, trnP(UGG), trnQ(UUG), trnR(ACG)^a^, trnR(UCU), trnS(GCU), trnS(GGA), trnS(UGA), trnT(GGU), trnT(UGU), trnV(UAC), trnV(GAC)^a^, trnW(CCA), trnY(GUA)

a Gene fully located within the inverted repeats.

b Gene partially located within the inverted repeats.

**Table 2:** Genes containing introns in the chloroplast genomes of *Stenogyne haliakalae* and 11 additional mint species. Numbers represent the lengths (bp) of exons and introns in *S. haliakalae*.

**Gene**	**Location**	**# Introns**	**Exon I**	**Intron I**	**Exon II**	**Intron II**	**Exon III**
atpF	LSC	1	143	656	410		
clpP	LSC	2	70	658	291	616	227
ndhA	SSC	1	552	1020	538		
ndhB	IR	1	755	680	776		
petB	LSC	1	5	718	650		
petD	LSC	1	7	728	474		
rpl16	LSC	1	8	907	392		
rpl2	IR	1	390	658	433		
rpoC1	LSC	1	434	736	1634		
rps12	LSC/IR^a^	2	113	a	231	537	25
rps16	LSC	1	39	875	226		
trnA-UGC	IR	1	37	807	34		
trnG-UCC	LSC	1	22	690	47		
trnI-GAU	IR	1	34	938	36		
trnK-UUU	LSC	1	36	2509	34		
trnL-UAA	LSC	1	36	488	49		
trnV-UAC	LSC	1	37	579	36		
ycf3	LSC	2	123	713	229	725	152

a Trans-spliced

**Table 3:** Lengths (bp) of the long single copy region (LSC), short single copy region (SSC), and inverted repeats regions (IR) for the chloroplast genomes of *Stenogyne haliakalae* and 11 additional mint species.

**Species**	**LSC**	**IR**	**SSC**	**Total**
*Haplostachys haplostachya*	81,755	25,441	17,495	150,132
*Haplostachys linearifolia*	81,752	25,441	17,495	150,129
*Phyllostegia velutina*	81,765	25,440	17,496	150,141
*Phyllostegia waimeae*	81,744	25,448	17,497	150,137
*Stachys byzantina*	81,245	25,480	17,517	149,722
*Stachys chamissonis*	81,774	25,464	17,552	150,254
*Stachys coccinea*	82,171	25,470	17,563	150,674
*Stachys sylvatica*	81,807	25,414	17,560	150,195
*Stenogyne bifida*	81,752	25,441	17,495	150,129
*Stenogyne haliakalae*	81,364	25,436	17,499	149,736
*Stenogyne kanehoana*	81,739	25,441	17,495	150,116
*Stenogyne sessilis*	81,743	25,441	17,495	150,120

**Table 4:** Sliding window analysis of variability of complete chloroplast genome sequences of three Hawaiian and three Stachys taxa (*Stenogyne haliakalae*, *Stenogyne bifida*, *Haplostachys haplostachya*, *Stachys chamissonis*, *Stachys coccinea*, and *Stachys sylvatica*). PICs – Potentially informative characters.

**Begin**	**End**	**# PICs*****Stachys***	**# PICs Hawaiian**
1	999	8	1
1000	1999	7	2
2000	2999	6	4
3000	3999	11	1
4000	4999	7	0
5000	5999	5	1
6000	6999	10	1
7000	7999	8	0
8000	8999	6	2
9000	9999	9	0
10,000	10,999	4	1
11,000	11,999	3	0
12,000	12,999	4	0
13,000	13,999	9	4
14,000	14,999	4	2
15,000	15,999	1	0
16,000	16,999	3	0
17,000	17,999	6	0
18,000	18,999	5	3
19,000	19,999	2	0
20,000	20,999	3	0
21,000	21,999	0	0
22,000	22,999	5	1
23,000	23,999	1	0
24,000	24,999	0	0
25,000	25,999	1	0
26,000	26,999	4	1
27,000	27,999	5	3
28,000	28,999	9	2
29,000	29,999	4	1
30,000	30,999	11	1
31,000	31,999	5	1
32,000	32,999	3	0
33,000	33,999	3	1
34,000	34,999	8	1
35,000	35,999	1	0
36,000	36,999	1	0
37,000	37,999	5	0
38,000	38,999	0	0
39,000	39,999	1	0
40,000	40,999	5	0
41,000	41,999	3	1
42,000	42,999	1	0
43,000	43,999	5	2
44,000	44,999	6	1
45,000	45,999	10	0
46,000	46,999	3	1
47,000	47,999	7	0
48,000	48,999	0	0
49,000	49,999	6	1
50,000	50,999	4	0
51,000	51,999	2	0
52,000	52,999	3	1
53,000	53,999	5	3
54,000	54,999	8	3
55,000	55,999	8	2
56,000	56,999	2	0
57,000	57,999	6	1
58,000	58,999	9	1
59,000	59,999	4	2
60,000	60,999	3	3
61,000	61,999	7	1
62,000	62,999	2	0
63,000	63,999	6	1
64,000	64,999	5	1
65,000	65,999	6	1
66,000	66,999	8	0
67,000	67,999	12	0
68,000	68,999	6	0
69,000	69,999	0	0
70,000	70,999	7	0
71,000	71,999	5	3
72,000	72,999	3	0
73,000	73,999	3	1
74,000	74,999	4	2
75,000	75,999	2	1
76,000	76,999	5	0
77,000	77,999	5	0
78,000	78,999	7	0
79,000	79,999	7	1
80,000	80,999	4	1
81,000	81,999	10	0
82,000	82,999	2	2
83,000	83,999	0	1
84,000	84,999	1	0
85,000	85,999	1	0
86,000	86,999	2	0
87,000	87,999	0	0
88,000	88,999	1	1
89,000	89,999	0	0
90,000	90,999	1	0
91,000	91,999	0	0
92,000	92,999	1	0
93,000	93,999	0	0
94,000	94,999	2	1
95,000	95,999	1	0
96,000	96,999	2	0
97,000	97,999	0	0
98,000	98,999	0	0
99,000	99,999	1	0
100,000	100,999	1	0
101,000	101,999	1	0
102,000	102,999	1	0
103,000	103,999	0	0
104,000	104,999	5	2
105,000	105,999	1	0
106,000	106,999	4	0
107,000	107,999	9	0
108,000	108,999	4	0
109,000	109,999	16	3
110,000	110,999	6	2
111,000	111,999	10	1
112,000	112,999	7	0
113,000	113,999	8	3
114,000	114,999	4	0
115,000	115,999	5	0
116,000	116,999	6	4
117,000	117,999	9	4
118,000	118,999	3	0
119,000	119,999	5	0
120,000	120,999	11	2
121,000	121,999	10	1
122,000	122,999	7	5
123,000	123,999	11	2
124,000	124,999	4	1
**Min**		0	0
**Max**		16	5
**Total**		565	104
**Mean**		4.52	0.83
**Median**		4.00	0.00

**Table 5:** Comparison of variability of coding genes by exon in complete chloroplast genome sequences of three Hawaiian and three *Stachys* taxa (*Stenogyne haliakalae, Stenogyne bifida, Haplostachys haplostachya, Stachys chamissonis, Stachys coccinea,* and *Stachys sylvatica*). Note: Exon numbers are defined by position in overall chloroplast genome sequence (not direction of gene) and exons that are completely conserved among these taxa and/or shorter than 100 bp have been excluded. PICs – Potentially informative characters.

**Region**	**Length**	**#PICs per locus*****Stachys***	**#PICs per locus Hawaiian**	**% PICs per locus*****Stachys***	**% PICs per locus Hawaiian**
psbA	1056	0	2	0.00	0.19
matK	1530	14	5	0.92	0.33
psbK	186	1	0	0.54	0.00
atpA	1524	5	1	0.33	0.07
atpF Exon1	411	1	0	0.24	0.00
atpI	744	1	0	0.13	0.00
rps2	711	1	0	0.14	0.00
rpoC2	4083	16	3	0.39	0.07
rpoC1 Exon1	1635	1	0	0.06	0.00
rpoC1 Exon2	435	1	0	0.23	0.00
rpoB	3213	4	0	0.12	0.00
psbD	1062	4	0	0.38	0.00
psbC	1422	3	1	0.21	0.07
psaB	2205	6	0	0.27	0.00
psaA	2253	2	0	0.09	0.00
rps4	606	1	0	0.17	0.00
ndhJ	477	3	0	0.63	0.00
ndhK	678	3	0	0.44	0.00
atpE	402	2	0	0.50	0.00
atpB	1497	4	0	0.27	0.00
rbcL	1446	12	6	0.83	0.41
accD	1467	3	0	0.20	0.00
ycf4	555	2	0	0.36	0.00
cemA	690	2	1	0.29	0.14
petA	963	2	1	0.21	0.10
rpl33	201	1	0	0.50	0.00
rpl20	387	2	0	0.52	0.00
psbB	1527	9	0	0.59	0.00
petB Exon2	651	2	1	0.31	0.15
rpoA	1014	3	1	0.30	0.10
rpl36	114	2	0	1.75	0.00
rps8	414	1	0	0.24	0.00
rpl14	369	2	0	0.54	0.00
rpl16 Exon1	393	3	0	0.76	0.00
rps3	663	3	0	0.45	0.00
rpl22	465	1	1	0.22	0.22
rps19	279	6	0	2.15	0.00
rpl2 Exon1	434	1	0	0.23	0.00
ycf2	6849	5	2	0.07	0.03
ndhB Exon1	756	1	0	0.13	0.00
rps7	468	2	1	0.43	0.21
rrn23	2811	1	0	0.04	0.00
ndhF	2229	16	0	0.72	0.00
rpl32	177	2	0	1.13	0.00
ccsA	970	3	2	0.31	0.21
ndhD	1521	11	1	0.72	0.07
psaC	244	1	0	0.41	0.00
ndhE	306	1	2	0.33	0.65
ndhG	531	1	0	0.19	0.00
ndhI	507	2	0	0.39	0.00
ndhA Exon1	539	3	0	0.56	0.00
ndhA Exon2	553	3	1	0.54	0.18
ycf1 SSC	4487	43	11	0.96	0.25
**Min**	114	0	0	0.00	0.00
**Max**	6849	43	11	2.15	0.65
**Total**	61110	225	43	23.43	3.45
**Mean**	1153	4.25	0.81	0.44	0.07
**Median**	678	2.00	0.00	0.33	0.00

**Table 6:** Comparison of variability of introns in complete chloroplast genome sequences of three Hawaiian and three Stachys taxa (Stenogyne haliakalae, Stenogyne bifida, Haplostachys haplostachya, Stachys chamissonis, Stachys coccinea, and Stachys sylvatica). Note: Exon and intron numbers are defined by position in the overall chloroplast genome sequence (not direction of gene). The trnK-UUU intron contains the gene matK. Here trnK-UUU-1 refers to the region between exon 1 and matK, and trnK-UUU-2 refers to the region between matK and exon2. PICs – Potentially informative characters.

**Region**	**Length**	**#PICs per locus*****Stachys***	**#PICs per locus Hawaiian**	**% PICs per locus*****Stachys***	**% PICs per locus Hawaiian**
trnK-UUU − 1	264	5	0	1.89	0.00
trnk-UUU − 2	714	5	0	0.70	0.00
rps16	874	5	1	0.57	0.11
trnG-UCC	689	7	0	1.02	0.00
atpF	655	3	0	0.46	0.00
rpoC1	762	4	1	0.52	0.13
ycf3-1	724	0	1	0.00	0.14
ycf3-2	712	2	0	0.28	0.00
trnL-UAA	487	4	0	0.82	0.00
trnV-UAC	478	1	1	0.21	0.21
clpP − 1	615	6	0	0.98	0.00
clpP − 2	657	4	0	0.61	0.00
petB	717	2	0	0.28	0.00
petD	727	4	2	0.55	0.28
rpl16	906	6	0	0.66	0.00
rpl2	657	2	2	0.30	0.30
trnI-GAU	937	1	0	0.11	0.00
ndhA	1019	10	7	0.98	0.69
**Min**	264	0	0	0.00	0.00
**Max**	1019	10	7	1.89	0.69
**Sum**	12594	71	15	10.95	1.86
**Mean**	699.7	3.94	0.83	0.61	0.10
**Median**	713	4.00	0.00	0.56	0.00

**Table 7:** Comparison of variability of intergenic spacers and pseudogenes in complete chloroplast genome sequences of three Hawaiian and three *Stachys* taxa (*Stenogyne haliakalae, Stenogyne bifida, Haplostachys haplostachya, Stachys chamissonis, Stachys coccinea,* and *Stachys sylvatica*). Note: Regions that are completely conserved among these taxa and/or shorter than 100 bp have been excluded. PICs – potentially informative characters.

**Region**	**Length**	**#PICs per locus*****Stachys***	**#PICs per locus Hawaiian**	**% PICs per locus*****Stachys***	**% PICs per locus Hawaiian**
trnH-GUG-psbA	323	8	1	2.48	0.31
psbA-trnK-UUU	244	2	0	0.82	0.00
trnK-UUU-rps16	643	5	0	0.78	0.00
rps16-trnQ-UUG	828	10	1	1.21	0.12
psbK-psbI	374	7	0	1.87	0.00
psbI-trnS-GCU	121	1	1	0.83	0.83
trnS-GCU-trnG-UCC	694	5	1	0.72	0.14
trnG-UCC-trnR-UCU	160	2	0	1.25	0.00
atpF-atpH	365	5	1	1.37	0.27
atpH-atpI	975	8	5	0.82	0.51
rps2-rpoC2	337	2	0	0.59	0.00
rpoC2-rpoC1	154	1	0	0.65	0.00
rpoB-trnC-GCA	1098	6	4	0.55	0.36
trnC-GCA-petN	436	4	0	0.92	0.00
petN-psbM	509	5	2	0.98	0.39
psbM-trnD-GUC	518	3	0	0.58	0.00
trnE-UUC-trnT-GGU	570	7	1	1.23	0.18
trnT-GGU-psbD	1075	8	1	0.74	0.09
psbC-trnS-UGA	232	3	1	1.29	0.43
trnS-UGA-psbZ	328	3	0	0.91	0.00
psbZ-trnG-GCC	224	2	0	0.89	0.00
trnG-GCC-trnfM-CAU	149	1	0	0.67	0.00
psaA-ycf3	730	7	0	0.96	0.00
ycf3-trnS-GGA	316	2	2	0.63	0.63
trnS-GGA-rps4	162	1	0	0.62	0.00
rps4-trnT-UGU	385	3	1	0.78	0.26
trnT-UGU-trnL-UAA	690	9	0	1.30	0.00
trnL-UAA-trnF-GAA	295	3	0	1.02	0.00
trnF-GAA-ndhJ	658	0	1	0.00	0.15
ndhC-trnV-UAC	944	5	0	0.53	0.00
trnV-UAC-trnM-CAU	188	1	0	0.53	0.00
trnM-CAU-atpE	213	2	0	0.94	0.00
atpB-rbcL	801	1	1	0.12	0.12
rbcL-accD	707	7	2	0.99	0.28
accD-psaI	397	1	1	0.25	0.25
psaI-ycf4	434	7	0	1.61	0.00
ycf4-cemA	306	5	1	1.63	0.33
cemA-petA	217	1	1	0.46	0.46
petA-psbJ	1059	9	3	0.85	0.28
psbE-petL	915	6	1	0.66	0.11
petL-petG	174	2	0	1.15	0.00
petG-trnW-CCA	125	1	0	0.80	0.00
trnP-UGG-psaJ	270	2	1	0.74	0.37
psaJ-rpl33	478	5	0	1.05	0.00
rpl33-rps18	146	0	1	0.00	0.68
rps18-rpl20	216	5	0	2.31	0.00
rpl20-rps12	739	10	0	1.35	0.00
psbB-psbT	184	1	1	0.54	0.54
psbH-petB	124	1	0	0.81	0.00
petB-petD	194	1	0	0.52	0.00
infA-rps8	123	1	0	0.81	0.00
rps8-rpl14	173	3	0	1.73	0.00
rpl14-rpl16	126	1	0	0.79	0.00
rpl16-rps3	141	1	1	0.71	0.71
rps12-3׳end-trnV-GAC	1448	3	0	0.21	0.00
trnA-UGC-rrn23	199	1	0	0.50	0.00
rrn4.5-rrn5	224	2	1	0.89	0.45
rrn5-trnR-ACG	234	2	1	0.85	0.43
trnR-ACG-trnN-GUU	569	2	0	0.35	0.00
ycf1Truncated	1059	1	0	0.09	0.00
ndhF-rpl32	435	5	2	1.15	0.46
rpl32-trnL-UAG	737	13	2	1.76	0.27
ccsA-ndhD	180	4	0	2.22	0.00
ndhD-psaC	105	1	0	0.95	0.00
psaC-ndhE	251	5	0	1.99	0.00
ndhG-ndhI	375	6	0	1.60	0.00
rps15-ycf1 SSC	389	3	0	0.77	0.00
**Min**	105	0	0	0.00	0.00
**Max**	1448	13	5	2.48	0.83
**Sum**	29192	250	43	62.72	10.44
**Mean**	435.7	3.73	0.64	0.94	0.16
**Median**	328.0	3.00	0.00	0.82	0.00

**Table 8:** Tailed primers used for multiplex amplification and targeted re-sequencing in mints**.** Sequences complimentary to the Illumina sequencing adapters were appended to the end of each primer (Forward: 5’ TCGTCGGCAGCGTCAGATGTGTATAAGAGACAG-[locus specific sequence] and Reverse: 5’ GTCTCGTGGGCTCGGAGATGTGTATAAGAGACAG-[locus specific sequence]). The loci within each region are also indicated. When the names of two genes are given with an underscore between them, the intergenic spacer between these genes is included in the amplified region.

**Primer**	**Sequence 5’ – 3’**	**Tm**	**Loci in region (excluding priming sites)**
Mint204F	TCG TCG GCA GCG TCA GAT GTG TAT AAG AGA CAG CGC CCC TCT ACT ATA ATG AAT GA	69.3	trnH-GUG_psbA
Mint204R	GTC TCG TGG GCT CGG AGA TGT GTA TAA GAG ACA GAC AGG ATC CAG AAA AAG AAA GA	68.1
Mint771F	TCG TCG GCA GCG TCA GAT GTG TAT AAG AGA CAG CCA TGA GCG GCT ACG ATA TT	70.3	psbA
Mint771R	GTC TCG TGG GCT CGG AGA TGT GTA TAA GAG ACA GCA GGC TGA GCA CAA CAT TCT	70.2
Mint867F	TCG TCG GCA GCG TCA GAT GTG TAT AAG AGA CAG GGA ACC ATG CAT AGC ACT GA	70.4	psbA
Mint867R	GTC TCG TGG GCT CGG AGA TGT GTA TAA GAG ACA GTA CCC AAT CGG TCA AGG AAG	69.1
Mint1170F	TCG TCG GCA GCG TCA GAT GTG TAT AAG AGA CAG CAC TCA CGA CCC ATG TAA CAA	70.1	psbA
Mint1170R	GTC TCG TGG GCT CGG AGA TGT GTA TAA GAG ACA GTG AGC CTG TTT CTG GAT CTC T	69.5
Mint1939F	TCG TCG GCA GCG TCA GAT GTG TAT AAG AGA CAG TCA AAA CAA AAG TTG AAT ACT CAG TTG	67.6	trnK-UUU Intron1, matK
Mint1939R	GTC TCG TGG GCT CGG AGA TGT GTA TAA GAG ACA GCG GAT TTG GTA TTT GGA TAT GA	68.3
Mint3998F	TCG TCG GCA GCG TCA GAT GTG TAT AAG AGA CAG GCT TCC CGT ATC AGG CAC T	71.2	trnk-UUU Intron2
Mint3998R	GTC TCG TGG GCT CGG AGA TGT GTA TAA GAG ACA GTC GAA TTC TTG GAA CGG AAC	68.6
Mint4546F	TCG TCG GCA GCG TCA GAT GTG TAT AAG AGA CAG CAG AAT TGT CAA AAT GTA TAG AGC A	68.2	trnK-UUU Exon2_rps16 Exon1
Mint4546R	GTC TCG TGG GCT CGG AGA TGT GTA TAA GAG ACA GCA TCG TGA TAA GCG ATC TGG	69.2
Mint4714F	TCG TCG GCA GCG TCA GAT GTG TAT AAG AGA CAG GAC CCA GAT CGC TTA TCA CG	70.1	trnK-UUU Exon2_rps16 Exon1
Mint4714R	GTC TCG TGG GCT CGG AGA TGT GTA TAA GAG ACA GTG TCA ATC CAA GAC AAT TTT GAA	67.8
Mint5533F	TCG TCG GCA GCG TCA GAT GTG TAT AAG AGA CAG TCC GAT CCA GTT ATT GAG ACG	69.0	rps16 Intron
Mint5533R	GTC TCG TGG GCT CGG AGA TGT GTA TAA GAG ACA GCG GGA ATC GAC TGT CCA TAG	69.7
Mint5985F	TCG TCG GCA GCG TCA GAT GTG TAT AAG AGA CAG GCC CCC GAG AAA TGA ATT A	69.9	rps16 Intron
Mint5985R	GTC TCG TGG GCT CGG AGA TGT GTA TAA GAG ACA GTA GAA AGC AAC GTG CGA CTT	69.3
Mint6787F	TCG TCG GCA GCG TCA GAT GTG TAT AAG AGA CAG TTC CAA TTT TGC ATT CGA GTC	68.5	rps16 Exon2_trnQ-UUG
Mint6787R	GTC TCG TGG GCT CGG AGA TGT GTA TAA GAG ACA GAA AAG GGT GAG TGG GTA GGA	69.7
Mint7334F	TCG TCG GCA GCG TCA GAT GTG TAT AAG AGA CAG TCA AAA ACG CAA CCA AAA TG	68.4	trnQ-UUG_psbK, psbK
Mint7334R	GTC TCG TGG GCT CGG AGA TGT GTA TAA GAG ACA GTG GCA TAA CAT CCA CGA TTG	68.8
Mint8052F	TCG TCG GCA GCG TCA GAT GTG TAT AAG AGA CAG GGT TTC CTT GGG TTT GGT TA	69.9	psbI-trnS-GCU, trnS-GCU
Mint8052R	GTC TCG TGG GCT CGG AGA TGT GTA TAA GAG ACA GGG AGA GAT GGC TGA GTG GAC	70.3
Mint8691F	TCG TCG GCA GCG TCA GAT GTG TAT AAG AGA CAG CCG AAC TCA AAA ATA AAC TGT CG	68.7	trnS-GCU_trnG-UCC Exon1
Mint8691R	GTC TCG TGG GCT CGG AGA TGT GTA TAA GAG ACA GCA AAA CGA GAA CGT TGC ACT	69.7
Mint8791F	TCG TCG GCA GCG TCA GAT GTG TAT AAG AGA CAG ATG AAG CCT CTT TCC CGA A	69.8	trnS-GCU_trnG-UCC Exon1, trnG-UCC Exon1
Mint8791R	GTC TCG TGG GCT CGG AGA TGT GTA TAA GAG ACA GCG GTT ACT AGA ACG AAT CAC ACT TT	68.9
Mint10381F	TCG TCG GCA GCG TCA GAT GTG TAT AAG AGA CAG TGC GCC AAT TCC AAT TTT A	69.0	atpA
Mint10381R	GTC TCG TGG GCT CGG AGA TGT GTA TAA GAG ACA GTC AAT CGG GAG ATG TTT CG	68.7
Mint10531F	TCG TCG GCA GCG TCA GAT GTG TAT AAG AGA CAG CAT TAA TGG CGG GTC TGA TT	69.9	atpA
Mint10531R	GTC TCG TGG GCT CGG AGA TGT GTA TAA GAG ACA GCT TTT GGA AAG AGC CGC TAA	69.6
Mint11293F	TCG TCG GCA GCG TCA GAT GTG TAT AAG AGA CAG CTG AGC AAT TCT TCC TGT TGC	69.9	atpA
Mint11293R	GTC TCG TGG GCT CGG AGA TGT GTA TAA GAG ACA GGG CGA TGG TAT TGC TCG TAT	69.8
Mint13441F	TCG TCG GCA GCG TCA GAT GTG TAT AAG AGA CAG CAG CAG CAA TAA CGG AAG C	70.3	atpH, atpH_atpI
Mint13441R	GTC TCG TGG GCT CGG AGA TGT GTA TAA GAG ACA GCG AAG TCG TTC TGA TGA TTC AA	68.8
Mint13731F	TCG TCG GCA GCG TCA GAT GTG TAT AAG AGA CAG AGC CAC GAC GAT ATG AAA GG	69.4	atpH_atpI
Mint13731R	GTC TCG TGG GCT CGG AGA TGT GTA TAA GAG ACA GAA AGT GGA TTG GTT GTC GAA	68.7
Mint14054F	TCG TCG GCA GCG TCA GAT GTG TAT AAG AGA CAG GAT TGA TCT AAG TTC ATG CAA TTT TT	67.8	atpH_atpI
Mint14054R	GTC TCG TGG GCT CGG AGA TGT GTA TAA GAG ACA GTT GTC CAC TTA ATA TCC TAC CTT TCC	67.9
Mint16824F	TCG TCG GCA GCG TCA GAT GTG TAT AAG AGA CAG TGA AAT CAA GAC CTT TGA TGT TAT T	67.7	rpoC2
Mint16824R	GTC TCG TGG GCT CGG AGA TGT GTA TAA GAG ACA GGA GGG TTG GAA CGA ACG TAT	69.8
Mint17255F	TCG TCG GCA GCG TCA GAT GTG TAT AAG AGA CAG CCG GAG TGG CCA AAT AAG T	70.8	rpoC2
Mint17255R	GTC TCG TGG GCT CGG AGA TGT GTA TAA GAG ACA GTT GGT ATT TTC TCC ATC CCA AT	68.3
Mint17928F	TCG TCG GCA GCG TCA GAT GTG TAT AAG AGA CAG TGG ATT TCA GCA AGT CGA TTC	68.9	rpoC2
Mint17928R	GTC TCG TGG GCT CGG AGA TGT GTA TAA GAG ACA GCC TTT ATG GAA ATG GGA AAC C	68.8
Mint20423F	TCG TCG GCA GCG TCA GAT GTG TAT AAG AGA CAG ATG CCA TTC CGA AGT GAT CT	69.6	rpoC2, rpoC2_rpoC1 Exon1
Mint20423R	GTC TCG TGG GCT CGG AGA TGT GTA TAA GAG ACA GCG CGA ATC AAG ATC GAG AAC	69.3
Mint20572F	TCG TCG GCA GCG TCA GAT GTG TAT AAG AGA CAG CGA TTT GAC AAT GGG TTT GA	69.4	rpoC2_rpoC1 Exon1, rpoC1 Exon1
Mint20572R	GTC TCG TGG GCT CGG AGA TGT GTA TAA GAG ACA GAA GCC ATA CAG GGG TTT TCC	69.4
Mint21910F	TCG TCG GCA GCG TCA GAT GTG TAT AAG AGA CAG GAG CTC ATT AAT TTA CCC CCA TC	69.0	rpoC1 Exon1
Mint21910R	GTC TCG TGG GCT CGG AGA TGT GTA TAA GAG ACA GGG GAA TGA ATG GGA AGA TCA	69.2
Mint22486F	TCG TCG GCA GCG TCA GAT GTG TAT AAG AGA CAG TCA AGT TAC CAG TGA AGA CTA AGC A	69.0	rpoC1 Intron
Mint22486R	GTC TCG TGG GCT CGG AGA TGT GTA TAA GAG ACA GGA TTC ATC ATT CGA AGG GAA GT	68.8
Mint23294F	TCG TCG GCA GCG TCA GAT GTG TAT AAG AGA CAG AAA TTC GAC TCC GCA TTG TT	69.2	rpoC1 Exon2
Mint23294R	GTC TCG TGG GCT CGG AGA TGT GTA TAA GAG ACA GGC CCA ATG GAG AGA TAG TCG	69.7
Mint23536F	TCG TCG GCA GCG TCA GAT GTG TAT AAG AGA CAG AAT CCA ATT CGG AGC TGT TG	69.1	rpoB
Mint23536R	GTC TCG TGG GCT CGG AGA TGT GTA TAA GAG ACA GTG GTC AAG TCA TAA AGT CAA ATA AA	67.2
Mint24088F	TCG TCG GCA GCG TCA GAT GTG TAT AAG AGA CAG GGC GCT ATT CGA TAA TGT CTG	69.3	rpoB
Mint24088R	GTC TCG TGG GCT CGG AGA TGT GTA TAA GAG ACA GGG AAG ACA CGG AWA CAA AGG	69.3
Mint24204F	TCG TCG GCA GCG TCA GAT GTG TAT AAG AGA CAG CAA CAG GTC TTC CAT CTT GC	69.9	rpoB
Mint24204R	GTC TCG TGG GCT CGG AGA TGT GTA TAA GAG ACA GCC GGG TTA TTG ATG TGA GGT	70.0
Mint27582F	TCG TCG GCA GCG TCA GAT GTG TAT AAG AGA CAG CTG ATT GGT TCA GTC TCA GTT TTT	69.1	rpoB_trnC-GCA
Mint27582R	GTC TCG TGG GCT CGG AGA TGT GTA TAA GAG ACA GGC GAG AGA ATG TTT TTA GCA TTG	68.4
Mint27884F	TCG TCG GCA GCG TCA GAT GTG TAT AAG AGA CAG GCA AAT CCT TTT TCC CCA GT	70.1	trnC-GCA, trnC-GCA_petN
Mint27884R	GTC TCG TGG GCT CGG AGA TGT GTA TAA GAG ACA GAA CGG GGC TTC ACA ATC TTT	69.5
Mint28672F	TCG TCG GCA GCG TCA GAT GTG TAT AAG AGA CAG AAG AGA TTC GGA TGA TTG GAA A	68.4	petN_psbM
Mint28672R	GTC TCG TGG GCT CGG AGA TGT GTA TAA GAG ACA GCG AGC GCA CTA TAA TCA GCA	69.9
Mint28881F	TCG TCG GCA GCG TCA GAT GTG TAT AAG AGA CAG GCT GAT TAT AGT GCG CTC GTT	70.0	petN_psbM, psbM
Mint28881R	GTC TCG TGG GCT CGG AGA TGT GTA TAA GAG ACA GTC CTA CCG CCT TTC TGC TTA	69.7
Mint30267F	TCG TCG GCA GCG TCA GAT GTG TAT AAG AGA CAG GGG GAG GAG TAG AAT CTC TTC A	69.9	trnE-UUC_trnT-GGU
Mint30267R	GTC TCG TGG GCT CGG AGA TGT GTA TAA GAG ACA GTT GTT TCA AGA CCA GCC CTA	69.3
Mint31916F	TCG TCG GCA GCG TCA GAT GTG TAT AAG AGA CAG GGG GTT GGT TCA CAG GTA CA	71.0	psbD
Mint31916R	GTC TCG TGG GCT CGG AGA TGT GTA TAA GAG ACA GCC ACC TAA TTG ACA CCA ACG	69.5
Mint32003F	TCG TCG GCA GCG TCA GAT GTG TAT AAG AGA CAG GGT CCT GAA GCA CAA GGA GA	70.9	psbD
Mint32003R	GTC TCG TGG GCT CGG AGA TGT GTA TAA GAG ACA GGC AAT TGG ACC AGA GAA TGC	69.6
Mint32348F	TCG TCG GCA GCG TCA GAT GTG TAT AAG AGA CAG TTC ATA ATT GGA CGC TGA ACC	68.9	psbD
Mint32348R	GTC TCG TGG GCT CGG AGA TGT GTA TAA GAG ACA GGC GGT CAC CAT GGA ATA AGT	70.0
Mint33303F	TCG TCG GCA GCG TCA GAT GTG TAT AAG AGA CAG GGG AGG GGG AGA TGT AAG AA	70.6	psbC
Mint33303R	GTC TCG TGG GCT CGG AGA TGT GTA TAA GAG ACA GGA TAT GCC AGA TTC CAC CAA G	69.1
Mint34416F	TCG TCG GCA GCG TCA GAT GTG TAT AAG AGA CAG GGG ATT CGA ACC CTC GAT A	70.1	trnS-UGA, trnS-UGA_psbZ
Mint34416R	GTC TCG TGG GCT CGG AGA TGT GTA TAA GAG ACA GAC CGG TCG GTA GAT TCA CAC	69.6
Mint35190F	TCG TCG GCA GCG TCA GAT GTG TAT AAG AGA CAG AAT GCG GAT ATG GTC GAA TG	68.9	trnG-GCC, trnG-GCC_trnfM_CAU
Mint35190R	GTC TCG TGG GCT CGG AGA TGT GTA TAA GAG ACA GGG TTT GGC TCT TAC CCC TTT	70.1
Mint35459F	TCG TCG GCA GCG TCA GAT GTG TAT AAG AGA CAG AGG ATT TGA ACC CGT GAC CT	70.2	trnfM-CAU, trnfM-CAU_rps14
Mint35459R	GTC TCG TGG GCT CGG AGA TGT GTA TAA GAG ACA GAC GGT CGA CGA TCA TAA AGG	68.9
Mint36179F	TCG TCG GCA GCG TCA GAT GTG TAT AAG AGA CAG CCA ATC TTG CTT GCA CAA TG	69.7	psaB
Mint36179R	GTC TCG TGG GCT CGG AGA TGT GTA TAA GAG ACA GCT GGG CGT GGA TGT TCT TAT	70.0
Mint36649F	TCG TCG GCA GCG TCA GAT GTG TAT AAG AGA CAG GTC CCA TGC CGA AAT ATC AC	69.7	psaB
Mint36649R	GTC TCG TGG GCT CGG AGA TGT GTA TAA GAG ACA GGC CTG GAG ACT TTT TGG TTC	69.5
Mint36907F	TCG TCG GCA GCG TCA GAT GTG TAT AAG AGA CAG CAT TGA GCA AAT ATG GGT TCG	69.2	psaB
Mint36907R	GTC TCG TGG GCT CGG AGA TGT GTA TAA GAG ACA GGA GAT TAC AAC CCG GAG CAA	69.9
Mint38366F	TCG TCG GCA GCG TCA GAT GTG TAT AAG AGA CAG TGC CAT AAT GCC TTT CAA ATC	68.6	psaB-psaA, psaA
Mint38366R	GTC TCG TGG GCT CGG AGA TGT GTA TAA GAG ACA GAT CCA TCG TTT GGG CTC AT	69.5
Mint41292F	TCG TCG GCA GCG TCA GAT GTG TAT AAG AGA CAG TAT TCG AAA CGC CTC GTG AT	69.5	ycf3 Exon1, ycf Intron1
Mint41292R	GTC TCG TGG GCT CGG AGA TGT GTA TAA GAG ACA GCG GTT CTA AGG GAA GGG ATT	69.9
Mint43421F	TCG TCG GCA GCG TCA GAT GTG TAT AAG AGA CAG TTG GAG CCT CGA AAG AAA GA	69.5	ycf3 Exon3_trnS-GGA
Mint43421R	GTC TCG TGG GCT CGG AGA TGT GTA TAA GAG ACA GAC TCG GCC ATC TCT CCT ACA	70.0
Mint47802F	TCG TCG GCA GCG TCA GAT GTG TAT AAG AGA CAG GAT GGC GTT TGA TAG AGG AAT C	69.1	ndhK
Mint47802R	GTC TCG TGG GCT CGG AGA TGT GTA TAA GAG ACA GTT GTC CAC CTA AAC CGG AAG	69.3
Mint50255F	TCG TCG GCA GCG TCA GAT GTG TAT AAG AGA CAG TTG GTA CCT AAA CGG GCA CT	70.3	trnV-UAC Intron, trnV-UAC Exon2
Mint50255R	GTC TCG TGG GCT CGG AGA TGT GTA TAA GAG ACA GTC AGT TGG TAG AGC ACC TCG T	70.0
Mint51642F	TCG TCG GCA GCG TCA GAT GTG TAT AAG AGA CAG TTC GGA TAA TTC GTC CAA CC	68.9	atpB
Mint51642R	GTC TCG TGG GCT CGG AGA TGT GTA TAA GAG ACA GTG CCA AAG GGA TCT ATC CAG	69.2
Mint51809F	TCG TCG GCA GCG TCA GAT GTG TAT AAG AGA CAG AGG ATC GGT CAA ATC GTC TG	69.3	atpB
Mint51809R	GTC TCG TGG GCT CGG AGA TGT GTA TAA GAG ACA GTC GAC AAT ATC TTC CGT TTC G	68.2
Mint52064F	TCG TCG GCA GCG TCA GAT GTG TAT AAG AGA CAG GGA AAT ATT CCG CCA TCG TT	69.8	atpB
Mint52064R	GTC TCG TGG GCT CGG AGA TGT GTA TAA GAG ACA GTA TCC GTA TTT GGC GGA GTG	69.1
Mint53793F	TCG TCG GCA GCG TCA GAT GTG TAT AAG AGA CAG GAC AAC TGT GTG GAC CGA TG	70.3	rbcL
Mint53793R	GTC TCG TGG GCT CGG AGA TGT GTA TAA GAG ACA GAG CAC GTA GGG CTT TGA ATC	69.3
Mint53955F	TCG TCG GCA GCG TCA GAT GTG TAT AAG AGA CAG AAA GCC CTA CGT GCT CTA CG	70.0	rbcL
Mint53955R	GTC TCG TGG GCT CGG AGA TGT GTA TAA GAG ACA GAG CAG ATA ACC CCA ATT TCG	68.7
Mint54300F	TCG TCG GCA GCG TCA GAT GTG TAT AAG AGA CAG TTT ATG CGT TGG AGA GAT CG	68.8	rbcL
Mint54300R	GTC TCG TGG GCT CGG AGA TGT GTA TAA GAG ACA GAT TGG CAG TGA ATC CTC CTG	69.3
Mint54974F	TCG TCG GCA GCG TCA GAT GTG TAT AAG AGA CAG GAC GTG ATC TTG CTG CTG AG	70.3	rbcL, rbcL_accD
Mint54974R	GTC TCG TGG GCT CGG AGA TGT GTA TAA GAG ACA GGA TTG GGC CGA GTT TAA TTG	68.9
Mint55144F	TCG TCG GCA GCG TCA GAT GTG TAT AAG AGA CAG CAA TTA AAC TCG GCC CAA TC	69.4	rbcL_accD
Mint55144R	GTC TCG TGG GCT CGG AGA TGT GTA TAA GAG ACA GTT GTG GAT CCA AGA CAC CAA	69.4
Mint55263F	TCG TCG GCA GCG TCA GAT GTG TAT AAG AGA CAG CGA AGA CTC CCA TTT TTC TCA	69.5	rbcL_accD
Mint55263R	GTC TCG TGG GCT CGG AGA TGT GTA TAA GAG ACA GGT CTA TTC CAA CAC GGA ACG A	69.5
Mint56316F	TCG TCG GCA GCG TCA GAT GTG TAT AAG AGA CAG CGC CCA CTG TAA GTG ATA GC	70.3	accD
Mint56316R	GTC TCG TGG GCT CGG AGA TGT GTA TAA GAG ACA GGC ATT GAA CCC ACA ACT GCT	70.4
Mint57465F	TCG TCG GCA GCG TCA GAT GTG TAT AAG AGA CAG TTT TTG AGT TCT ACA TTC CTT GGA C	68.2	accD_psaI
Mint57465R	GTC TCG TGG GCT CGG AGA TGT GTA TAA GAG ACA GTG GGT ACC TCG ATT TAC TAT TTG T	68.2
Mint58710F	TCG TCG GCA GCG TCA GAT GTG TAT AAG AGA CAG TGA TGA GAA TTT GAC TCC ACG A	69.0	ycf4, ycf4_cemA
Mint58710R	GTC TCG TGG GCT CGG AGA TGT GTA TAA GAG ACA GTG TGA TGA TCA AAA AGT CGA TTG	67.7
Mint59661F	TCG TCG GCA GCG TCA GAT GTG TAT AAG AGA CAG TCC GTC ACT TGT AGT TAT TTA TCA TTC	67.6	cemA, cemA_petA
Mint59661R	GTC TCG TGG GCT CGG AGA TGT GTA TAA GAG ACA GTT CCC TGT CCA AGA TTC TGC	69.3
Mint60529F	TCG TCG GCA GCG TCA GAT GTG TAT AAG AGA CAG AGA TTT ATC CCG ACG GAA GC	69.4	petA
Mint60529R	GTC TCG TGG GCT CGG AGA TGT GTA TAA GAG ACA GTT GAT GGA TTC ACC CTC TGA	69.1
Mint60945F	TCG TCG GCA GCG TCA GAT GTG TAT AAG AGA CAG CGG ATT TAT GGA CAT CAG GTT	69.5	petA_psbJ
Mint60945R	GTC TCG TGG GCT CGG AGA TGT GTA TAA GAG ACA GTG GTC AAG TCA TAA AGT CAA ATA AA	67.2
Mint61133F	TCG TCG GCA GCG TCA GAT GTG TAT AAG AGA CAG GAC TTG ACC ACC CCC TTC TT	71.0	petA_psbJ
Mint61133R	GTC TCG TGG GCT CGG AGA TGT GTA TAA GAG ACA GAT TCT TTG TCC CAC GCA TTC	68.8
Mint62265F	TCG TCG GCA GCG TCA GAT GTG TAT AAG AGA CAG CCC CCA GTA GAG ACT GGT ACG	71.0	psbL, psbL_psbF, psbF
Mint62265R	GTC TCG TGG GCT CGG AGA TGT GTA TAA GAG ACA GAG TAC GCT GGT TGG CTG TTC	70.0
Mint64488F	TCG TCG GCA GCG TCA GAT GTG TAT AAG AGA CAG AAA ACA AAC GCG CTA CCA AG	69.4	trnP-UGG, trnP-UGG_psaJ
Mint64488R	GTC TCG TGG GCT CGG AGA TGT GTA TAA GAG ACA GCC AAT TGA AAT GTA AAA CGC TCT	68.6
Mint65276F	TCG TCG GCA GCG TCA GAT GTG TAT AAG AGA CAG TTT ACT ATG GCT TTG CTT TGA TTT	68.0	psaJ_rpl33, rpl33
Mint65276R	GTC TCG TGG GCT CGG AGA TGT GTA TAA GAG ACA GTT CCA AAA TCA CCG TTA CCC	68.8
Mint65764F	TCG TCG GCA GCG TCA GAT GTG TAT AAG AGA CAG CGG GGG ATC GAA TTG ATT AT	69.6	rps18
Mint65764R	GTC TCG TGG GCT CGG AGA TGT GTA TAA GAG ACA GGG TCG ACT CGA TTC TTT CAA AT	68.8
Mint68295F	TCG TCG GCA GCG TCA GAT GTG TAT AAG AGA CAG TCT CTC GAT ACA TAA TCG AAT CTT TT	67.5	clpP Intron1, clpP Exon2
Mint68295R	GTC TCG TGG GCT CGG AGA TGT GTA TAA GAG ACA GTG GTT GGA GGA GAA ATT ACC A	69.0
Mint68800F	TCG TCG GCA GCG TCA GAT GTG TAT AAG AGA CAG CCT TTT GGT GCA TAC GGT TC	70.1	clpP Intron2
Mint68800R	GTC TCG TGG GCT CGG AGA TGT GTA TAA GAG ACA GCC ATC GTG ATT TGG ATT GAA	68.9
Mint71208F	TCG TCG GCA GCG TCA GAT GTG TAT AAG AGA CAG ATC GTG CGA CTT TGA AAT CC	69.1	psbB
Mint71208R	GTC TCG TGG GCT CGG AGA TGT GTA TAA GAG ACA GCC CAA GTT TTT GGA ATG CTC	69.2
Mint71701F	TCG TCG GCA GCG TCA GAT GTG TAT AAG AGA CAG CGA GAA CCA CCT AAA GTT CCA	70.0	psbT, psbT_psbN, psbN
Mint71701R	GTC TCG TGG GCT CGG AGA TGT GTA TAA GAG ACA GCC GGG TAC GCC TTA TAT ACC	69.6
Mint72221F	TCG TCG GCA GCG TCA GAT GTG TAT AAG AGA CAG GAC TCC TTT GAT GGG TGT CG	70.2	psbH, psbH_petB Exon1
Mint72221R	GTC TCG TGG GCT CGG AGA TGT GTA TAA GAG ACA GGG CCG CAA ATT TGA GTT CTA	69.5
Mint73489F	TCG TCG GCA GCG TCA GAT GTG TAT AAG AGA CAG TTG ACT TGG GTT ACG GGT GT	70.3	petB Exon2
Mint73489R	GTC TCG TGG GCT CGG AGA TGT GTA TAA GAG ACA GCG AGT CAA GGT GGA TTG TCC	70.0
Mint74004F	TCG TCG GCA GCG TCA GAT GTG TAT AAG AGA CAG TTA TGG GAG TGT GCG ACT TG	69.6	petD Intron
Mint74004R	GTC TCG TGG GCT CGG AGA TGT GTA TAA GAG ACA GCG GAG CCT ACT CAT GTA CAA C	69.5
Mint74125F	TCG TCG GCA GCG TCA GAT GTG TAT AAG AGA CAG CAG AAG ATG GGC TGG TTC AC	70.6	petD Intron
Mint74125R	GTC TCG TGG GCT CGG AGA TGT GTA TAA GAG ACA GAA TGC AGA GGA AAT GAA TGC	68.3
Mint75956F	TCG TCG GCA GCG TCA GAT GTG TAT AAG AGA CAG CGA CAT AAG GCG GTA AGA TGA	69.9	rpoA
Mint75956R	GTC TCG TGG GCT CGG AGA TGT GTA TAA GAG ACA GTG AGA ATG TCC CGC ATG AAT	69.4
Mint76974F	TCG TCG GCA GCG TCA GAT GTG TAT AAG AGA CAG TCT GCG GAT TAG TCG ACA TTT	69.3	rpl36
Mint76974R	GTC TCG TGG GCT CGG AGA TGT GTA TAA GAG ACA GCC GAA ACA AGG ATT CGA AAG	68.9
Mint79374F	TCG TCG GCA GCG TCA GAT GTG TAT AAG AGA CAG ATT GCT TTC CGG TTC ATT TC	68.6	rpl16 Intron
Mint79374R	GTC TCG TGG GCT CGG AGA TGT GTA TAA GAG ACA GCA AGA GCT TCG AGC CAA TAA	69.4
Mint79823F	TCG TCG GCA GCG TCA GAT GTG TAT AAG AGA CAG AAT ATG AAG CGA TGG GTT GG	69.0	rpl16 Intron, rpl16 Exon2, rpl16 Exon2_rps3
Mint79823R	GTC TCG TGG GCT CGG AGA TGT GTA TAA GAG ACA GTG GCC AAT CAA ACA AAT TCC	68.6
Mint80282F	TCG TCG GCA GCG TCA GAT GTG TAT AAG AGA CAG TGC CTG TTC AGT CAA TTC AA	69.2	rps3
Mint80282R	GTC TCG TGG GCT CGG AGA TGT GTA TAA GAG ACA GCG CGA GGA ATC GAA GAA TTA	69.1
Mint80833F	TCG TCG GCA GCG TCA GAT GTG TAT AAG AGA CAG TAG CCC GGG GTT TTA ATT TC	69.2	rpl22
Mint80833R	GTC TCG TGG GCT CGG AGA TGT GTA TAA GAG ACA GCC GTT CCT ACG AGG AAA CAC	69.9
Mint106808F	TCG TCG GCA GCG TCA GAT GTG TAT AAG AGA CAG TGG ACC CGT TTC TGA AGA GT	70.1	ycf1Truncated, ycf1Truncated_ndhF, ndhF
Mint106808R	GTC TCG TGG GCT CGG AGA TGT GTA TAA GAG ACA GTT TTT GGA GAA GGG ATC AAA	68.1
Mint107075F	TCG TCG GCA GCG TCA GAT GTG TAT AAG AGA CAG CCC ATC GTT TTC TTT TTG GA	69.4	ndhF
Mint107075R	GTC TCG TGG GCT CGG AGA TGT GTA TAA GAG ACA GGG ATT CGG CAA GTT GGT ATG	69.4
Mint107435F	TCG TCG GCA GCG TCA GAT GTG TAT AAG AGA CAG TCG TAT TGG CGG ATT CAT AA	68.8	ndhF
Mint107435R	GTC TCG TGG GCT CGG AGA TGT GTA TAA GAG ACA GGG GGT AAA GGG TAT TCC AAA A	69.1
Mint107714F	TCG TCG GCA GCG TCA GAT GTG TAT AAG AGA CAG TGA ATG TTT AAA TGC CCC TCA	69.0	ndhF
Mint107714R	GTC TCG TGG GCT CGG AGA TGT GTA TAA GAG ACA GAG GTA CAC TTT CGC TTT GTG G	69.1
Mint108575F	TCG TCG GCA GCG TCA GAT GTG TAT AAG AGA CAG CGC TTT TTG ACA AGC ATT TG	69.1	ndhF
Mint108575R	GTC TCG TGG GCT CGG AGA TGT GTA TAA GAG ACA GTG GCT CAC GAT CAA GGA TAC	69.1
Mint109649F	TCG TCG GCA GCG TCA GAT GTG TAT AAG AGA CAG GTA TCG GGC AGC GTT AAA AG	69.7	rpl32, rpl32_trnL-UAG
Mint109649R	GTC TCG TGG GCT CGG AGA TGT GTA TAA GAG ACA GAT TCC CCG TTG AAG GAA ATG	68.8
Mint110066F	TCG TCG GCA GCG TCA GAT GTG TAT AAG AGA CAG TCT CGC TAT CAA TCC ACA CAA	69.2	rpl32_trnL-UAG
Mint110066R	GTC TCG TGG GCT CGG AGA TGT GTA TAA GAG ACA GTA GAA CCC TCC CTC CCC AAA	70.4
Mint110450F	TCG TCG GCA GCG TCA GAT GTG TAT AAG AGA CAG GGT AGA CAC GCT GCT CTT AGG	70.4	trnL-UAG, trnL-UAG_ccsA, ccsA
Mint110450R	GTC TCG TGG GCT CGG AGA TGT GTA TAA GAG ACA GTC GAA ACG ATC GAA AAA GAA	67.9
Mint111053F	TCG TCG GCA GCG TCA GAT GTG TAT AAG AGA CAG CTA TGC GGC CCT TTT ATG TG	70.0	ccsA
Mint111053R	GTC TCG TGG GCT CGG AGA TGT GTA TAA GAG ACA GTT GGA ATT CAC CAA CGA AAA	68.3
Mint113090F	TCG TCG GCA GCG TCA GAT GTG TAT AAG AGA CAG GGA TCA TCC GAT TGA AAA TGA	68.8	ndhD
Mint113090R	GTC TCG TGG GCT CGG AGA TGT GTA TAA GAG ACA GAC GAA CCA TTT TCC TTG CTT	68.9
Mint113960F	TCG TCG GCA GCG TCA GAT GTG TAT AAG AGA CAG TTC AGC GGC TGC AAT AGT TA	69.7	ndhE
Mint113960R	GTC TCG TGG GCT CGG AGA TGT GTA TAA GAG ACA GTG CCT ATT TAT TTT CCA TTG GT	67.9
Mint116500F	TCG TCG GCA GCG TCA GAT GTG TAT AAG AGA CAG CCG TTA CCG TCG CTA TTA CAG	69.9	ndhA Intron
Mint116500R	GTC TCG TGG GCT CGG AGA TGT GTA TAA GAG ACA GGC AGA CAG AAT TCC ATT GGT C	69.2
Mint116861F	TCG TCG GCA GCG TCA GAT GTG TAT AAG AGA CAG TTG CAA TTC TCG TTT TTG GA	68.7	ndhA Intron
Mint116861R	GTC TCG TGG GCT CGG AGA TGT GTA TAA GAG ACA GGA ATT GGG GCT TTA AGT TGG T	69.4
Mint116914F	TCG TCG GCA GCG TCA GAT GTG TAT AAG AGA CAG TGG TGG ATA GGA ACA TAC TCT GG	69.3	ndhA Intron
Mint116914R	GTC TCG TGG GCT CGG AGA TGT GTA TAA GAG ACA GTG GAT GGT TAG GAA GAC CAA A	69.0
Mint117269F	TCG TCG GCA GCG TCA GAT GTG TAT AAG AGA CAG CAG CAA AAT TTT AAG CCG TTT T	68.9	ndhA Intron
Mint117269R	GTC TCG TGG GCT CGG AGA TGT GTA TAA GAG ACA GCG TGT GAT TCG GTG AGA CAT	69.9
Mint117433F	TCG TCG GCA GCG TCA GAT GTG TAT AAG AGA CAG TGT CTC ACC GAA TCA CAC GTA	69.7	ndhA Exon2
Mint117433R	GTC TCG TGG GCT CGG AGA TGT GTA TAA GAG ACA GCG CCG ATC TTA GTA TTG GTG T	69.6
Mint119376F	TCG TCG GCA GCG TCA GAT GTG TAT AAG AGA CAG TTT TGA CAA ATA GGC CAG CA	69.3	rps15
Mint119376R	GTC TCG TGG GCT CGG AGA TGT GTA TAA GAG ACA GTG CAT CGA TTT CGG TTA TTT C	68.0
Mint122192F	TCG TCG GCA GCG TCA GAT GTG TAT AAG AGA CAG CAT TTC TTG GTT TTC GAA TTT TT	67.9	ycf1 single copy
Mint122192R	GTC TCG TGG GCT CGG AGA TGT GTA TAA GAG ACA GGC ATC TCC GAG TTG GAC AAA	70.0
Mint122492F	TCG TCG GCA GCG TCA GAT GTG TAT AAG AGA CAG AAA AAC ACT TTT GAG AAC CCA TTT	68.2	ycf1 single copy
Mint122492R	GTC TCG TGG GCT CGG AGA TGT GTA TAA GAG ACA GAA CCG AAC TCC CCT TTT GTT	69.4
Mint122638F	TCG TCG GCA GCG TCA GAT GTG TAT AAG AGA CAG TTT TTC GGG GTG AAC AAA AG	68.8	ycf1 single copy
Mint122638R	GTC TCG TGG GCT CGG AGA TGT GTA TAA GAG ACA GTG GTT GAC GGA TGG TAT TCA	69.1
Mint123159F	TCG TCG GCA GCG TCA GAT GTG TAT AAG AGA CAG TTT GAA ATG CTT CCC CCT TA	69.2	ycf1 single copy
Mint123159R	GTC TCG TGG GCT CGG AGA TGT GTA TAA GAG ACA GCG GCA CGG TAT AAT CAA AGG	69.4
Mint124145F	TCG TCG GCA GCG TCA GAT GTG TAT AAG AGA CAG TTC GAT TAT AGG CGG GGA TA	69.1	ycf1 single copy
Mint124145R	GTC TCG TGG GCT CGG AGA TGT GTA TAA GAG ACA GAT CGC TGG AAT CGA CCA TTT	69.3
