# Supplementary material for: Data characterizing the chloroplast genomes of extinct and endangered Hawaiian endemic mints (Lamiaceae) and their close relatives
Source: Data Brief. 2016 Mar 14;7:900–22. doi: 10.1016/j.dib.2016.03.037 (PMC4816906; doi:10.1016/j.dib.2016.03.037)
Supplement: Supplementary file 1 — Supplementary material [file mmc1.docx]

**Supplementary Information**

Welch AJ, Collins K, Ratan A, Drautz-Moses DI, Schuster SC, Lindqvist C. 2016. ***Data characterizing the chloroplast genomes of extinct and endangered Hawaiian endemic mints (Lamiaceae)* *and their close relatives*.** Data in Brief.

**Supplementary Table 1.** Primers used for closing gaps between assembled contigs and for refinement of the reference *Stenogyne haliakalae* sequence.

| **Primer** | **Sequence (5'-3')** | **Ta** |
| --- | --- | --- |
| AssemblyContig_A-BF | GGGAAGGTTCTTCGTATCTATCA | 50 |
| AssemblyContig_A-BR | TGAATTTTAATATCTGTGTCTGTTCG | 50 |
| AssemblyContig_B-CF | TTTGTTTTTGTTCCAACGAATT | 50 |
| AssemblyContig_B-CR | GATTCGGGAAAGAGGCTTCA | 50 |
| AssemblyContig_C-DF | CCGATTCCACAAATCTTTCG | 50 |
| AssemblyContig_C-DR | TTGGATGATAGACCAGAACAATC | 50 |
| AssemblyContig_D-EF | ATGCTTAGCCAATTCCATGC | 50 |
| AssemblyContig_D-ER | CTACCCAGGGCTTCGATACA | 50 |
| AssemblyContig_E-FF | CGGTATTGGGGAGACAAAGA | 50 |
| AssemblyContig_E-FR | TGGAACCGGCAATAAAAGAG | 50 |
| AssemblyContig_F-GF | CCCGCCCTTGTATTGTGTAT | 50 |
| AssemblyContig_F-GR | CGAGCGCACTATAATCAGCA | 50 |
| AssemblyContig_G-HF | CCTTTTTGCTTTGGGAATGA | 50 |
| AssemblyContig_G-HR | TTCAGAAAGACCCCACGAAG | 50 |
| AssemblyContig_H-IF | TGAATCGTTCATTAGAATCGACA | 50 |
| AssemblyContig_H-IR | TTGTTTCAAGACCAGCCCTAA | 50 |
| AssemblyContig_I-JF | TTTGTCGATATTTCCGATTCAA | 50 |
| AssemblyContig_I-JR | CGGATGCGGCAATATAGTTT | 50 |
| AssemblyContig_J-KF | GTCCCAGATCCAAAATGACC | 50 |
| AssemblyContig_J-KR | ATCGGACCGGAGACTCTCTT | 50 |
| AssemblyContig_K-LF | CTTTGGTTGGCTCTTTCCAC | 50 |
| AssemblyContig_K-LR | CGAGGCGTTTCGAATAGAAG | 50 |
| AssemblyContig_L-MF | ATCGTTGTTTTGGACGATGC | 50 |
| AssemblyContig_L-MR | TATGGCCGTGATCTGTCATT | 50 |
| AssemblyContig_M-NF | CTTCCGCATAATTTCCTTCG | 50 |
| AssemblyContig_M-NR | GCAGGAAGGTTGGCTAGAGA | 50 |
| AssemblyContig_N-N2F | TCATTCCCTTTGATCTCACAAA | 50 |
| AssemblyContig_N-N2R | TCGAGGCTCCAAGATAAAGAG | 50 |
| AssemblyContig_N2-OF | TTTTGTTTACCGAGGGTTCG | 50 |
| AssemblyContig_N2-OR | ATTGCTAGTCGATCCGGTGT | 50 |
| AssemblyContig_O-PF | TTGCTAAAGAAAGCGACTTCAA | 50 |
| AssemblyContig_O-PR | GAATCGTTTAAAGCCGAACG | 50 |
| AssemblyContig_P-QF | CGAGGTGCTCTACCAACTGA | 50 |
| AssemblyContig_P-QR | CACCACTCAACCACTCCAAA | 50 |
| AssemblyContig_O-RF | AAGGTTCCCATCAAACTAGTGC | 50 |
| AssemblyContig_O-RR | TGGATGGATGATGAATAAGGA | 50 |
| AssemblyContig_R-SF | TGGATTCGATTGGATGCAC | 50 |
| AssemblyContig_R-SR | TTCGGGTTCGAATTCCATAG | 50 |
| AssemblyContig_S-TF | CGCCTATTCCTTTCTTTTCG | 50 |
| AssemblyContig_S-TR | TGTTCTGATCGCCAACTCAT | 50 |
| AssemblyContig_T-UF | TGAGAAAAGGATTGGGCTGA | 50 |
| AssemblyContig_T-UR | TGTGATGATCAAAAAGTCGATTG | 50 |
| AssemblyContig_U-VF | GTTTCGGGTTTCCAGGTGTA | 50 |
| AssemblyContig_U-VR | CACCTCTTCCTCGATCTTGA | 50 |
| AssemblyContig_V-WF | CCCGGGAGGTAGAGTAGGAA | 50 |
| AssemblyContig_V-WR | AAACCCGAAGGACATCAGTT | 50 |
| AssemblyContig_W-XF | TGCACCAGTGGAAACCATAA | 50 |
| AssemblyContig_W-XR | CAAAGCAAAGCCATAGTAAAGAA | 50 |
| AssemblyContig_X-YF | TTCCTTTCTTTTTCTTCATTTCG | 50 |
| AssemblyContig_X-YR | CAAGCCATTCGATCCTATTCA | 50 |
| AssemblyContig_Y-ZF | CACTGGTGCAAATCCAATCA | 50 |
| AssemblyContig_Y-ZR | AGGGTGTATGTGCGACTCGT | 50 |
| AssemblyContig_Z-AAF | GGGAAGTCCGTAGGATAGTCA | 50 |
| AssemblyContig_Z-AAR | AATGCAGAGGAAATGAATGC | 50 |
| AssemblyContig_AA-BBF | GAAAGTGCATCCTATTCCATGA | 50 |
| AssemblyContig_AA-BBR | GGGGGTCGTATTTCTTCTTATC | 50 |
| AssemblyContig_BB-CCF | AACATCGTATTGGCGGATTC | 50 |
| AssemblyContig_BB-CCR | TGGGGTAAAGGGTATTCCAA | 50 |
| AssemblyContig_CC-DDF | AATTCCCGAATTCCATTTCC | 50 |
| AssemblyContig_CC-DDR | TAGAACCCTCCCTCCCCAAA | 50 |
| AssemblyContig_DD-EEF | GCAATAAAAGGGTGTACAAGGT | 50 |
| AssemblyContig_DD-EER | ACAAAGAGCCCATCGAATCA | 50 |
| AssemblyContig_EE-FFF | CGTGGGTGATTTGGATTGAT | 50 |
| AssemblyContig_EE-FFR | GAACAATTTCTTCGAATTTACCTCA | 50 |
| AssemblyContig_FF-GGF | TTCCATCCCCAAAAACCATA | 50 |
| AssemblyContig_FF-GGR | CTCCCGGATAATTCAAATCG | 50 |
| AssemblyContig_GG-HHF | TTCCCTCTCTTTTGACCGATT | 50 |
| AssemblyContig_GG-HHR | ATGAGGAGCCGTATGAGGTG | 50 |
| AssemblyContig_HH-IIF | TTTGACCATTAGCATCAGTTACA | 50 |
| AssemblyContig_HH-IIR | TGAGCAAAAATCATTATCAACAG | 50 |
| AssemblyContig_II-JJF | ACGTTCTGAACCCAGCTCAC | 50 |
| AssemblyContig_II-JJR | ATCTTCCCCAAGAGCTCACA | 50 |
| AssemblyContig_JJ-KKF | TTCGATTGATGGGATTTGGT | 50 |
| AssemblyContig_JJ-KKR | CGCTTATGGGGTCAAATCAA | 50 |
| AssemblyContig_KK-AF | TTGTTCGTCGCCGTAGTAAA | 50 |
| AssemblyContig_KK-AR | GGGGGATTCTTAAGGATTGA | 50 |
| Mint9260F | TCCTCTCAAATAGAAATAGAGAACGA | 50 |
| Mint9260R | TTCATTCGGCTCCTTTATGG | 50 |
| Mint27185F | AATAGCCCTGGCTGTGCTC | 50 |
| Mint27185R | AAACTGAGACTGAACCAATCAGTA | 50 |
| Mint30196F | AGGGCTGGTCTTGAAACAAT | 50 |
| Mint30196R | CGGATTTGAACCGATGACTT | 50 |
| Mint44294F | TGAAGCGAAGTTTGCTGAAG | 50 |
| Mint44294R | AAAGTGTCTTCCCTTCTTTCCA | 50 |
| Mint57220F | TTTGAGTTCTACATTCCTTGGAC | 50 |
| Mint57220R | CATGAAGAAATAAAGAAGCCATTG | 50 |
| Mint64600F | CGGATGCGTTGACATTTCC | 50 |
| Mint64600R | TACCTCCCGGGCCTCTATTC | 50 |
| Mint71370F | TCGAATTTATGGAAGCATTGG | 50 |
| Mint71370R | AGATCCATTCGAGGAACACG | 50 |
| MintIRbF | TCAATGACAATCAGGATAATTCAAG | 50 |
| MintSSCR | GGAGTTGGCCCAATTAACG | 50 |
